# Outcomes of implantable cardioverter‐defibrillator implantation in HIV‐infected patients: A single‐center retrospective cohort study

**DOI:** 10.1002/clc.23868

**Published:** 2022-06-01

**Authors:** Venkata A. Narla, Hannan Yang, Quefeng Li

**Affiliations:** ^1^ Section of Cardiac Electrophysiology, Division of Cardiology, Department of Medicine University of North Carolina at Chapel Hill Chapel Hill North Carolina USA; ^2^ Department of Biostatistics, Gillings School of Public Health University of North Carolina at Chapel Hill Chapel Hill North Carolina USA

**Keywords:** HIV, implantable cardioverter‐defibrillators, sudden cardiac death

## Abstract

**Background:**

HIV‐infected individuals have a known increased risk of sudden cardiac death (SCD) compared to uninfected individuals. Implantable cardioverter‐defibrillators (ICDs) are standard therapy for preventing SCD; however, there is limited data on the outcomes of ICDs in HIV‐infected individuals.

**Hypothesis:**

HIV‐infected subjects receive a higher number of appropriate ICD therapies than uninfected controls.

**Methods:**

This is a retrospective cohort study of 35 consecutive HIV‐Infected patients and 36 uninfected controls matched by age, race, and gender who were treated at the University of North Carolina Medical Center in the outpatient or inpatient setting from 2014 to the present and had undergone ICD implantation. For HIV‐infected subjects, a multivariate Poisson regression analysis was performed to evaluate the association between covariates and ICD therapies.

**Results:**

Among HIV‐infected subjects, the mean CD4 count was 582.5 cells/mm^3^ and 69% had an undetectable viral load. The median follow‐up was 6.4 years. HIV‐infected subjects had both a higher number of appropriate ICD shocks or antitachycardia pacing (ATP) therapy per person‐year as well as a higher number of inappropriate ICD shocks per person‐year than uninfected controls (1.512 vs. 0.590 and 0.122 vs. 0.0166, respectively, *p* < .001 for both comparisons). After multivariate adjustment, the presence of detectable/unsuppressed viral load at the time of ICD implantation was an independent predictor of both of the following in HIV‐infected subjects: (1) appropriate ICD discharge (*p* = .004), and (2) appropriate ICD discharge or appropriate ATP therapy (*p* < .001).

**Conclusion:**

HIV‐infected subjects had a higher number of appropriate ICD discharge or ATP therapy per person‐year than matched uninfected controls.

## 1 INTRODUCTION

As a result of antiretroviral therapy (ART), HIV‐infected patients are living longer, and therefore, chronic diseases such as cardiovascular disease (CVD) are increasing in prevalence in this patient population.^1^ HIV‐infected individuals have higher rates of CVD than uninfected individuals likely due to a complex interaction of multiple possible factors such as side effects of ART, HIV‐associated inflammation, and increased prevalence of traditional risk factors among HIV‐infected individuals including cigarette smoking, hypertension, and dyslipidemia.^2^ Multiple studies of HIV‐infected patients have also revealed an increased prevalence of QT prolongation, a predictor of cardiovascular mortality.^3^, ^4^, ^5^, ^6^ Up to a 4.5‐fold increased risk of sudden cardiac death (SCD) has been demonstrated in HIV‐infected patients as compared to uninfected individuals.^7^

Implantable cardioverter‐defibrillators (ICDs) are standard therapy for preventing sudden arrhythmic death; however, there is limited data on the outcomes of ICDs in HIV‐infected individuals despite the increased risk of SCD among HIV‐infected patients. Furthermore, ICD data provides an objective surrogate for risk of sudden arrhythmic death, which would otherwise be presumed in studies without ICD data and/or autopsy data. Alvi et al.^8^ demonstrated that among HIV‐infected subjects with concomitant heart failure, rates of both appropriate and inappropriate ICD shock were higher than in uninfected controls. Additionally, ICD discharge was associated with increased cardiovascular mortality amongst both HIV‐infected and uninfected controls with heart failure.^8^ However, this study was limited to only subjects who had been admitted to the hospital for decompensated heart failure, which may represent a higher‐risk subgroup.

Further studies are needed to investigate the outcomes of ICD implantation in HIV patients who have not been admitted for decompensated heart failure. Therefore, our study seeks to address this gap by investigating the outcomes of ICD implantation in HIV patients who were followed at UNC Medical Center in the outpatient and inpatient setting with or without heart failure. Additionally, our single‐center study allows for comprehensive evaluation of electronic medical records including EKG predictors and ICD data when necessary. We hypothesize that HIV‐infected patients have a higher rate of appropriate ICD discharges for ventricular arrhythmias than uninfected controls given the higher risk of sudden arrhythmic death in HIV‐infected individuals.

## 2 METHODS

### 2.1 Study design and study population

After Institutional Review Board (IRB) approval was obtained from the University of North Carolina at Chapel Hill's IRB, data were requested from the Carolina Data Warehouse for Health, which is a centralized repository of clinical and research patient data sourced from the UNC Health Care System. This is a retrospective cohort study. We investigated among consecutive HIV‐ Infected patients and uninfected controls treated at UNC Medical Center in the outpatient or inpatient setting from 2014 to the present who underwent primary or secondary prevention ICD implantation, whether HIV‐infected patients have a higher number of appropriate ICD discharges or antitachycardia pacing (ATP) therapy per person‐year than uninfected controls. Uninfected controls with ICDs were matched by age, race, and gender in a 1:1 ratio. In addition to the data extracted from the Carolina Data Warehouse, any missing variables of interest were added to the dataset via a comprehensive chart review of individual electronic medical records.

### 2.2 Variables studied

Baseline demographic variables were collected. Additionally, covariates of interest for which data were collected via both comprehensive medical record review as well as from the Carolina Data Warehouse for Health included the following: HIV status, history of illicit drug use, history of alcohol abuse, left ventricular ejection fraction at the time of ICD implantation, history of coronary artery disease, dialysis, CD4 count at the time of ICD implantation, HIV viral load at the time of ICD Implantation, QRS duration at the time of ICD implantation, corrected QT (QTc) interval duration at the time of ICD implantation, whether or not the patient is on antiarrhythmics for secondary prevention of ventricular tachycardia (VT), and indication for ICD implantation (primary vs. secondary prevention).

For HIV‐specific features, electrocardiographic parameters, and echocardiographic data (CD4 count, HIV viral load, QRS duration, QTc duration, left ventricular ejection fraction), data were recorded from the time of ICD implantation or closest to the time of ICD implantation if otherwise unavailable despite comprehensive electronic medical record review.

### 2.3 Outcomes adjudication (ICD therapies)

Patients were followed from the time of ICD implantation to the present or until they were lost to follow‐up from the UNC Health Care System. Outcomes of interest included the percentage of individuals with appropriate ICD discharge or appropriate ATP therapy as well as the rate of appropriate ICD or ATP therapy per person‐year. Appropriate ICD shock or ATP therapy was defined as delivery of therapy for sustained VT or ventricular fibrillation. Inappropriate therapy was defined as shock or ATP delivered for supraventricular arrhythmia or for farfield oversensing and noise. ICD data were reviewed via electronic medical records by board‐certified electrophysiologist.

### 2.4 Statistical analysis

Data are presented as means ± standard deviation for continuous variables and as percentages for categorical variables. Baseline characteristics of subjects within each group were compared by two‐sample *t*‐test with unequal variances for continuous variables and by Fisher's exact test for categorical variables. Two‐tailed *p* < .05 was considered statistically significant. To compare the proportion of appropriate ICD discharges between HIV‐infected patients and uninfected controls, a two‐by‐two contingency table was constructed, and a logistic regression was used to compare if the proportions significantly differed between the two groups. To compare the number of appropriate ICD discharges per person‐year between HIV‐infected patients and uninfected controls, Poisson regression with time duration as an offset was used to compare if the numbers of appropriate ICD discharges per person‐year significantly differed between the two groups. All tests were two‐tailed with a significance level of *p* < .05. For HIV‐positive subjects, univariate and multivariate Poisson regression analyses were performed to evaluate the association between covariates and outcomes of interest (appropriate ICD discharge per person‐year in HIV‐positive individuals; appropriate ICD or ATP therapy per person year in HIV‐positive individuals). Covariates that were clinically relevant and with nonzero counts were incorporated into the multivariate Poisson regression analysis. They included the following predictors: (1) history of illicit drug use, (2) left ventricular ejection fraction less than 35%, (3) dialysis, (4) CD4 count greater than 200 copies/ml, (5) detectable/unsuppressed viral load, (6) on antiarrhythmics for VT, (7) secondary and primary prevention ICD indications, and (8) history of coronary artery disease. CD4 count was included as a binary variable into the model (<200 copies/ml or ≥200 copies/ml) as was the viral load (suppressed vs. unsuppressed/detectable). The statistical software R (version 3.6.3) was used to perform the analyses.

## 3 RESULTS

### 3.1 Sample characteristics

The study group included 35 HIV‐infected patients with ICDs who were followed within the UNC Health Care System from 2014 until the present and 36 uninfected patients with ICDs who were matched by age, race, and gender. Baseline characteristics are shown in Table 1. Among the HIV‐infected subjects, the mean CD4 count was 582.5 cells/mm^3^ and 69% had an undetectable viral load. Subjects with and without HIV did not differ significantly with respect to age, sex, race, left ventricular ejection fraction, and prevalence of coronary artery disease (Table 1). There was a trend toward a longer QTc interval at the time of ICD implantation in HIV‐infected subjects compared to uninfected controls (469.7 ± 35.8 vs. 454.7 ± 35.7 ms, *p* = .089, Table 1) but this was not statistically significant. 88.2% of HIV‐infected subjects had a primary prevention indication for ICD implantation while 72.2% of uninfected subjects had a primary prevention indication for ICD implantation.

**Table 1:** Baseline characteristics of HIV‐positive subjects with ICD versus HIV‐negative controls with ICD

Baseline variables	HIV‐positive subjects with ICD (*n* = 35)	HIV‐negative controls with ICD (*n* = 36)	*p*
Age – yr. mean (SD)	58.80 (11.67)	58.42 (12.44)	.894
Male sex – no. (%)	29 (82.9)	31 (86.1)	.959
Race or ethnic group – no. (%)			.403
White	10 (30.3)	8 (22.2)	
Black	22 (66.7)	28 (77.8)	
Other			
(American–Indian/Alaska Native)	1 (3.0)	0 (0)	
H/o illicit drug use – no. (%)	4 (11.4)	1 (2.8)	.337
H/o alcohol abuse – no. (%)	0 (0)	0 (0)	N/A
LVEF, % ±SD	28.16 (12.42)	32.08 (14.36)	.227
H/o CAD – no. (%)	24 (68.6)	18 (50)	.177
Dialysis – no. (%)	4 (11.8)	2 (5.6)	.617
CD4 cell count/mm^3^ mean (SD)	582.5 (384.28)		
Viral load undetectable – no. (%)	24 (68.6)		
QRS duration (ms)	118.48 (24.03)	111.97 (28.2)	.308
QTc duration (ms)	469.69 (35.78)	454.69 (35.69)	.089
On antiarrhythmics for VT – no. (%)	8 (22.9)	11 (20.6)	.642
Primary prevention ICD – no. (%)	31 (88.2)	26 (72.2)	.169
Secondary prevention ICD – no. (%)	4 (11.8)	10 (27.8)	.169
Death – no. (%)	5 (14.3)	9 (25)	.403

*Note*: Values shown are no. (%) or mean ± SD.

Abbreviations: CAD, coronary artery disease; H/o, history of; ICD, implantable cardioverter‐defibrillator; LVEF, left ventricular ejection fraction; N/A, not applicable; QTc, corrected QT interval; VT, ventricular tachycardia.

### 3.2 ICD discharge and ATP therapy

The median follow‐up was 6.4 years. Among the HIV‐infected subjects, 37.1% received an appropriate ICD shock or appropriate ATP therapy while 30.6% of uninfected subjects received an appropriate ICD shock or appropriate ATP therapy (*p* = .558, Table 2). 11.4% of HIV‐infected subjects had an inappropriate ICD discharge while 5.6% of uninfected controls had an inappropriate ICD discharge (*p* = .383, Table 2). HIV‐infected subjects had a higher number of appropriate ICD discharge or ATP therapy per person‐year than uninfected controls (1.512 vs. 0.590, *p* < .001, Table 2). HIV‐infected subjects also had a higher number of inappropriate ICD shocks per person‐year than uninfected controls (0.122 vs. 0.0166, *p* < .001). There was no significant association found between the number of appropriate ICD discharges per person‐year and mortality in HIV‐infected subjects (*p* = .18).

**Table 2:** ICD therapy in HIV‐positive subjects and HIV‐negative controls

**ICD treatment**	**HIV‐positive subjects with ICD**	**HIV‐negative controls with ICD**	***p***
% of individuals who had appropriate ICD discharge or ATP	37.14	30.56	.558
% of individuals who had appropriate ICD discharge only	31.43	25.00	.548
% of individuals who had inappropriate ICD discharge	11.43	5.56	.383
Number of appropriate ICD shock or ATP per person‐year	1.512	0.590	<.001*
Number of appropriate ICD shocks only per person‐year	0.194	0.133	.102
Number of inappropriate ICD shocks per person‐year	0.122	0.0166	<.001*

*Note*: Data are given as % or *n* as indicated.

Abbreviations: ATP, antitachycardia pacing; ICD, implantable cardioverter‐defibrillator.

* *p*‐value is statistically significant.

Univariate and multivariate Poisson regression analysis was performed to identify predictors of appropriate ICD discharge among HIV‐infected subjects. After multivariate adjustment, the presence of detectable/unsuppressed viral load at the time of ICD implantation and being on antiarrhythmics for treatment of VT were independent predictors of appropriate ICD discharge in HIV‐infected subjects (Table 3). There was no significant association between a history of illicit drug use and the number of inappropriate ICD discharges per person‐year both in HIV‐infected and uninfected subjects (*p* > .9 for both groups).

**Table 3:** Multivariable Poisson regression analysis of predictors of appropriate ICD discharge in HIV‐positive subjects

Variables^a^	Multivariate analysis	
	Incidence per person‐year (95% CI)	*p*
History of (H/o) illicit drug use	2.56 (0.28, 23.72)	.407
LVEF <35%	0.300 (0.047, 1.900)	.201
Dialysis	0.883 (0.182, 4.276)	.877
CD4 count >200	0.487 (0.041, 5.739)	.567
Viral load detectable	4.105 (1.562, 10.790)	.004*
On antiarrhythmics for VT	52.33 (12.20, 224.40)	<.001*
Secondary prevention ICD indication	0.20 (0.01, 3.85)	.286

Abbreviations: CI, confidence interval; ICD, implantable cardioverter‐defibrillator; LVEF, left ventricular ejection fraction; VT, ventricular tachycardia.

^a^ Of note, among HIV‐positive subjects without H/o coronary artery disease, there were zero patients who had an appropriate ICD shock. Therefore, H/o coronary artery disease was not included as a predictor in the above multivariate Poisson regression model since statistical comparison cannot be made.

* *p*‐value is statistically significant.

Univariate and multivariate Poisson regression analysis was also performed to identify predictors of appropriate ICD discharge *or* appropriate ATP therapy among HIV‐infected subjects. On multivariate Poisson analysis, the presence of detectable/unsuppressed viral load at the time of ICD implantation, secondary prevention indication for ICD implantation, and being on antiarrhythmics for treatment of VT remained independent predictors of appropriate ICD discharge *or* appropriate ATP therapy in HIV‐infected subjects (Table 4). Additionally, on multivariate analysis, a history of illicit drug use was associated with fewer appropriate ICD discharges or appropriate ATP therapies compared to those without a history of illicit drug use (Table 4).

**Table 4:** Multivariable Poisson regression analysis of predictors of appropriate ICD discharge or ATP therapy in HIV‐positive subjects

Variables	Multivariate analysis	
	Incidence per person‐year (95% CI)	*p*
H/o illicit drug use	0.237 (0.059, 0.948)	.042*
LVEF <35%	1.53 (0.273, 8.582)	.629
H/o CAD	1.46 (0.350, 6.086)	.604
Dialysis	0.53 (0.123, 2.261)	.388
CD4 count >200	0.58 (0.129, 2.577)	.471
Viral load detectable	3.91 (1.818, 8.397)	<.001*
On antiarrhythmics for VT	9.44 (2.758, 32.31)	<.001*
Secondary prevention ICD indication	16.7 (2.07, 142.9)	.009*

Abbreviations: ATP, antitachycardia pacing; CAD, coronary artery disease; CI, confidence interval; H/o, history of; ICD, implantable cardioverter‐defibrillator; LVEF, left ventricular ejection fraction; VT, ventricular tachycardia.

* *p*‐value is statistically significant.

## 4 DISCUSSION

In this study, we investigated HIV‐infected patients and uninfected controls treated at UNC Medical Center in the outpatient or inpatient setting since 2014 until present who underwent primary or secondary prevention ICD implantation. Our key findings can be summarized as follows: (1) HIV‐infected patients had both a higher number of appropriate ICD shocks or ATP per person‐year as well as a higher number of Inappropriate ICD shocks per person‐year than uninfected controls. (2) On multivariate analysis, presence of detectable/unsuppressed viral load at time of ICD implantation was an independent predictor of appropriate ICD discharge in HIV‐infected subjects. (3) On multivariate analysis, the presence of detectable/unsuppressed viral load at the time of ICD implantation, secondary prevention indication for ICD implantation, and being on antiarrhythmics for treatment of VT remained independent predictors of appropriate ICD discharge or appropriate ATP therapy in HIV‐infected subjects. History of illicit drug use was also associated with fewer appropriate ICD discharge or appropriate ATP therapy compared to those without a history of illicit drug use.

The percentage of individuals in this study who had appropriate and inappropriate ICD shocks is comparable to that found in other large‐scale studies. Saxon et al.^9^ found that among 194 000 patients in a prospective observational cohort, the appropriate and inappropriate shock incidence after 5 years of follow‐up was 23% and 17%, respectively. In other randomized controlled trials, the inappropriate shock rate had also been demonstrated to range anywhere from 10% to 24%.^10^, ^11^ Similarly, in our study, 28% of all individuals had an appropriate ICD shock and 8% of all individuals had an inappropriate ICD shock.

In our study, we found a significantly increased number of appropriate ICD shocks or ATP therapy per person‐year in HIV‐infected individuals as compared to uninfected controls. This finding supports other studies with respect to increased risk of sudden arrhythmic death in HIV‐infected individuals as compared to uninfected controls since appropriate ICD shock or appropriate ATP therapy for sustained ventricular arrhythmias can be considered a surrogate for sudden arrhythmic death.^7^ The mechanisms for this increased risk in this particular patient population are unclear but are likely multifactorial. Possible hypothesized mechanisms for the increased SCD risk in the HIV‐infected population include the following but are not limited to: substance abuse (cocaine and amphetamine use), accelerated atherosclerosis, chronic systemic inflammation, QTc prolongation, and myocardial fibrosis/scar.^2^

There was no significant difference in follow‐up time between the HIV‐infected patients and uninfected controls. While the percentage of individuals who had appropriate ICD discharge or ATP therapy did not significantly differ between the HIV‐infected patients and uninfected controls, the number of appropriate ICD shocks or ATP therapy per‐person year did significantly differ between the two groups (Table 2). This may suggest that even if the overall follow‐up time does not significantly differ between the two groups, it is possible that those HIV‐positive patients who had appropriate ICD shocks and ATP therapy may have been more likely to have repeated sustained ventricular arrhythmias throughout their followup time over multiple years given the presence and severity of their risk factors more so than HIV‐negative patients. This again alludes to the possible increased cumulative sudden arrhythmic death risk in HIV‐positive patients over their entire followup period as compared to HIV‐negative patients.

We also found in our study that detectable/unsuppressed viral load at the time of ICD implantation was an independent predictor of appropriate ICD therapy in HIV‐infected patients on multivariate analysis. This was also an independent predictor of appropriate ICD shock or appropriate ATP therapy in HIV‐infected patients also on multivariate analysis. To our knowledge, our study is one of the first to demonstrate this finding with respect to ICD outcomes. The mechanism of how unsuppressed viral load can lead to increased risk of sustained ventricular arrhythmias is not fully understood but could be due to a combination of the aforementioned mechanisms.^12^ The unsuppressed viral load may lead to an increased effect of HIV on repolarization and QTc interval, increased risk of premature atherosclerosis, increased risk of HIV‐associated cardiomyopathy, and increased myocardial fibrosis/scar due to systemic inflammation. HIV‐infected individuals have been shown to have an increased prevalence of myocardial fibrosis.^13^ Among 90 HIV‐infected individuals and 39 age‐matched controls, Holloway et al.^13^ found that myocardial fibrosis as measured by magnetic resonance imaging T1‐mapping and late gadolinium enhancement was present in 76% of the HIV‐infected subjects as compared to only 13% of the uninfected controls (*p* < .001). However, they did not find an association between recent viral load and the degree of myocardial fibrosis.^13^

In our study, there was a trend toward a longer baseline QTc interval among HIV‐infected individuals compared to uninfected controls (469.7 ± 35.8 vs. 454.7 ± 35.7 ms, *p* = .089) but this was not statistically significant. QT prolongation in HIV‐infected individuals may be seen as a result of both the direct effect of HIV viral infection on repolarization as well as due to concomitant administration of ARTs and other potentially QT‐prolonging medications, such as antibiotics, antifungals, antidepressants/antipsychotics, and methadone.^3^, ^4^, ^6^, ^14^, ^15^, ^16^ Studies with a larger sample size may allow for examination of whether QTc prolongation is an independent predictor of appropriate ICD discharge in this particularly vulnerable patient population.

In our study, those with a history of illicit drug use had fewer appropriate ICD discharges or appropriate ATP therapy compared to those without a history of illicit drug use after multivariate adjustment. Underreporting or underdocumentation of illicit drug use is a possible explanation for this finding. Furthermore, there was no significant association between a history of illicit drug use and the number of inappropriate ICD discharges per person year both in HIV‐infected and uninfected subjects. Alvi et al.^8^ had found that cocaine use was associated with increased ICD discharge in HIV‐infected individuals (appropriate and inappropriate ICD discharges were combined in this outcome). It is well‐known that cocaine use can lead to myocardial ischemia/infarction, sympathetic surges, and the development of cardiomyopathy, all potential mechanisms for contributing to the development of life‐threatening ventricular arrhythmias.^17^, ^18^ Larger studies are needed to investigate this possible association with ICD outcomes.

Our study has limitations. Given the limited number of HIV‐positive patients with ICDs who are typically followed within a single healthcare system, the small sample size in our study could potentially lead to the detection of a spurious correlation from the multivariate model. Further studies with a larger sample size may lend additional support and corroboration to our significant findings in this study. Furthermore, although our single‐center retrospective cohort study allows for a comprehensive and accurate evaluation of each patient's medical records, results may not be generalizable to other regions and settings. In addition, ICD programming was left to the discretion of the individual oupatient electrophysiologist providers who were following the patients given that this is a retrospective cohort study. Heterogeneity due to lack of ICD programming standardization cannot be ruled out. Lastly, causality cannot be inferred nor established as with all cohort studies, and the influence of potential hidden confounders cannot be ruled out. An important strength of our study is that ICD data provides a clear objective surrogate for sudden arrhythmic death and sustained ventricular arrhythmias. Without ICD data and/or autopsy data, SCD is only presumed in most studies.

In conclusion, this study demonstrated a significantly higher number of appropriate ICD shocks or ATP therapy per person‐year in HIV‐infected subjects compared to uninfected controls matched by age, race, and gender. Presence of detectable/unsuppressed viral load at the time of ICD implantation was an independent predictor of both of the following outcomes in HIV‐infected subjects: (1) appropriate ICD discharge, and (2) appropriate ICD discharge or appropriate ATP therapy. There was a trend toward longer baseline QTc interval among HIV‐infected individuals compared to uninfected controls but this was not statistically significant. ICD shocks can not only cause significant psychological distress but can also lead to increased mortality. Our data suggest that healthcare providers could focus on preventing ICD shocks amongst HIV‐infected subjects by controlling HIV viral load. Additionally, our data emphasize monitoring for QTc prolongation in HIV‐infected patients. Further prospective studies are needed to corroborate these findings.

## AUTHOR CONTRIBUTIONS

Venkata A. Narla was responsible for the concept, study design, acquisition of data, drafting of the manuscript, and critical revision of the manuscript. Hannan Yang and Quefeng Li were responsible for data analysis and statistics, and assisted in the revision of the manuscript.

## CONFLICT OF INTEREST

The authors declare no conflict of interest.

## Data Availability

Data are available on request.
